# Validation of outlier loci through replication in independent data sets: a test on *Arabis alpina*

**DOI:** 10.1002/ece3.1300

**Published:** 2014-10-24

**Authors:** Dominique Buehler, Rolf Holderegger, Sabine Brodbeck, Elvira Schnyder, Felix Gugerli

**Affiliations:** 1 WSL Swiss Federal Research InstituteZürcherstrasse 111, CH-8903, Birmensdorf, Switzerland; 2 Department of Environmental Systems Science, ETH ZürichUniversitätsstrasse 16, CH-8092, Zürich, Switzerland

**Keywords:** Allele distribution model, environmental association analysis, genome scan, natural selection, SNaPshot®

## Abstract

Outlier detection and environmental association analysis are common methods to search for loci or genomic regions exhibiting signals of adaptation to environmental factors. However, a validation of outlier loci and corresponding allele distribution models through functional molecular biology or transplant/common garden experiments is rarely carried out. Here, we employ another method for validation, namely testing outlier loci in specifically designed, independent data sets. Previously, an outlier locus associated with three different habitat types had been detected in *Arabis alpina*. For the independent validation data set, we sampled 30 populations occurring in these three habitat types across five biogeographic regions of the Swiss Alps. The allele distribution model found in the original study could not be validated in the independent test data set: The outlier locus was no longer indicative of habitat-mediated selection. We propose several potential causes of this failure of validation, of which unaccounted genetic structure and technical issues in the original data set used to detect the outlier locus were most probable. Thus, our study shows that validating outlier loci and allele distribution models in independent data sets is a helpful tool in ecological genomics which, in the case of positive validation, adds confidence to outlier loci and their association with environmental factors or, in the case of failure of validation, helps to explain inconsistencies.

## Introduction

Environmental association analysis (Holderegger et al. 2010; Schoville et al. 2012) has become a standard method to detect loci or genomic regions that are potentially under divergent selection caused by environmental factors. In a usual environmental association study, genome scans of samples from many locations and comprising hundreds to thousands of amplified fragment length polymorphisms (AFLPs) or thousands to millions of single nucleotide polymorphisms (SNPs) are first tested for signals of divergent selection, for example, among many other methods (Schoville et al. 2012), using outlier analysis (Vasemägi and Primmer 2005; Stinchcombe and Hoekstra 2008; Galindo et al. 2011). Subsequently, allele frequencies at these outlier loci are related to environmental factors such as precipitation, temperature, altitude, bedrock, soil, or habitat type (Manel et al. 2012; Buehler et al. 2013; Fischer et al. 2014); in other words, one establishes an allele distribution model that reveals which allele occurs under certain environmental conditions (Holderegger et al. 2008). Thus, the result of an environmental association analysis consists of a set of outlier loci of potential adaptive relevance to particular environmental factors. Whether these loci, or a locus physically linked to the respective outlier locus, are really under selection by a given environmental factor is, however, not proven at this stage of the analysis.

Outlier analysis and environmental association analysis often detect false positives, that is, loci that are considered to be under divergent selection by a given environmental factor while in fact they are not. Simulation studies have shown that the power and consistency of outlier detection methods perform rather poorly in this respect and that many false positives are usually detected (Vilas et al. 2012, De Mita et al. 2013; Lotterhos and Whitlock 2014). This leads to an important question: What are the causes for false positives? First and most prominently, geographic genetic structure conflicts with the detection of signals of adaptive divergence among populations (Excoffier et al. 2009; Shikano et al. 2010). Such genetic structure may arise through neutral processes that are mainly driven by migration, for example, in the course of postglacial recolonization of formerly unoccupied ranges resulting in distinct phylogeographic structure. Second, other demographic processes mainly acting at the local level, such as genetic drift, bottlenecks, inbreeding, or gene conversions, can interfere with signals of selection (Teshima et al. 2006; Stinchcombe and Hoekstra 2008; Excoffier et al. 2009; Buerkle et al. 2011). Third, purely coincidental spatial covariance of local environmental conditions and genetic variation can cause signals of selection and associations of alleles with environmental factors independent of selective processes (Shikano et al. 2010). Fourth, the type of genetic marker could also be considered a problem. For instance, AFLPs can be problematic in locus-specific analyses (Meudt and Clarke 2007). This multitude of interfering factors or processes often cannot be strictly distinguished as being the ultimate cause of neutral genetic structure. Therefore, a detected set of outlier loci, which might be of adaptive relevance to particular environmental factors, is prone to comprise false positives and hence needs further validation (Holderegger et al. 2008; Schoville et al. 2012).

Several ways how to validate outlier loci have been described. Most studies attempt to prove that the detected loci are potentially under selection by confirming that the respective outlier loci are located within, are closely linked to or at least lie in genomic regions that contain a gene of known function (but see Pavlidis et al. 2012). In an ideal case, this should fit the suggested function from environmental association analysis. In other words, researchers try to establish the link between the mechanistic function of genetic polymorphisms at or near an outlier locus with the respective environmental variation (Fischer et al. 2013). However, this is a formidable task and most studies stop somewhere in the middle of their way to this goal. Outlier loci or the genomic regions in which outlier loci are located have to be sequence-characterized. If the study is performed using SNP outliers from whole-genome sequencing, this step is straightforward. If the study applied random markers such as AFLPs or restriction site associated DNA (RAD) sequencing, additional laboratory and bioinformatic work are required (Baird et al. 2008; Minder and Widmer 2008; Zulliger et al. 2013). Sequences obtained are then compared to known genes from model organisms and to the, ideally, known molecular and ecological functions of these genes. For instance, in species related to the model plant *Arabidopsis thaliana*, a GO-Term analysis will accomplish this task (Primmer et al. 2013). However, in species that are not closely related to model organisms, such information on gene function is often unavailable. Although the comparison with known genes and their function may substantiate the findings that a particular outlier locus is under environmental selection, it does not yet provide a final proof of adaptive relevance. For this aim, further molecular and experimental work is needed that could include genetic engineering (*e.g.,* knock-out variants; Nordborg and Weigel 2008).

An additional way to validate outlier loci is to set up reciprocal transplant or common garden experiments (Reusch and Wood 2007; Holderegger et al. 2008). In these experiments, individuals carrying alleles associated with particular environmental conditions are grown under identical or contrasting conditions and their performance or fitness is measured. For instance, in an alpine plant species at locus XY, there might be allele b that is related to low temperatures and allele c that is related to higher temperatures. In a reciprocal transplant experiment, individuals with allele b should perform better at higher altitudes with prevailing low temperatures than individuals with allele c and *vice versa*. It is clear that in such transplant or common garden experiments, only the adaptive relevance of genes and alleles can be identified which have a strong effect on performance and fitness. In addition, such experiments are evidently difficult to carry out for many animal species, such as most vertebrates. It is therefore not surprising that, to the best of our knowledge, such transplant and common garden experiment to validate the adaptive relevance of outlier loci have hardly been carried out to date.

Another way to validate the adaptive relevance of outlier loci, which is strongly linked to ecological thinking, is to replicate results in a follow-up study. The idea being that once the association between environmental factors and allele frequencies has been established, new independent and specifically designed data sets are created to test the allele distribution model (Holderegger et al. 2008). In the above example, one could collect and genetically analyze many samples along several (*i.e.,* replicated) altitudinal gradients to validate the temperature-related fitness effects of alleles b and c at locus XY. However, independent data sets to validate outlier loci and the signals of selection across different regions and populations are rarely available (Wiener et al. 2011). Nevertheless, researchers have long carried out similar tests, deliberately or accidentally, when they transferred suggested allele distribution models from one study area (or laboratory) to another area (or the natural conditions). One study is the textbook example on coat color in pocket mice. Nachmann et al. (2003) found that coat color variation in pocket mice depends on ground type in dune systems and was associated with a single-gene mutation found in one population. However, the mutation did not show the same environmental association in a replicated population. Similarly, Korves et al. (2007) had difficulties to extend the relationship between season, flowering time, and alleles at the *FRIGIDA* gene, well known for its effect on life cycle and flowering behavior from laboratory experiments, to more than hundred natural accessions of *A. thaliana* across Europe. Such conflicting results point to the importance of critical validation of the ecological and adaptive relevance of outlier loci and corresponding allele distribution models.

**Figure 1:**
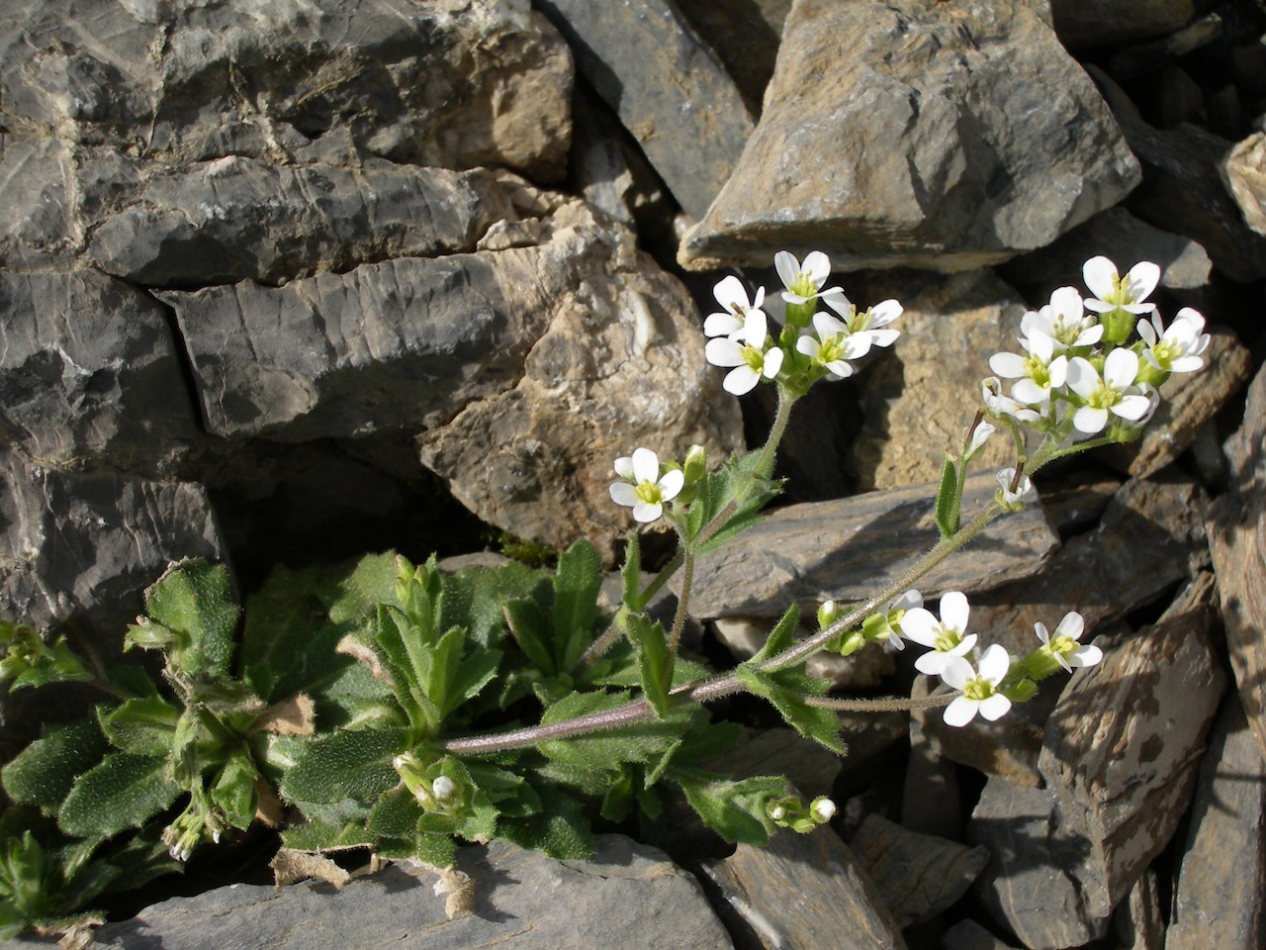
*Arabis alpina*, a widespread arctic-alpine Brassicaceae.

In this study, we argue that testing the generality of environmental association of alleles at outlier loci through validation in independent data sets could become a standard method in ecological genomics, especially as the aim of this field is to find ecological functions of genomic patterns (Reusch and Wood 2007). We illustrate this claim with an example of the alpine plant *Arabis alpina,* a widespread member of the Brassicaceae family (Fig. 1). In a previous study, we found allele frequencies at an AFLP outlier locus in *A. alpina* to be associated with different habitat types (Buehler et al. 2013). In this study, we first developed a specifically designed sample set in Switzerland to attempt a replication of the above result, avoiding locations already sampled by Buehler et al. (2013). Next, we genetically screened hundreds of sampled individuals in a fast and labor-effective SNP assay and finally tested whether the originally found allele distribution model was confirmed and thus validated in the new independent data set.

## Materials and Methods

### Original allele distribution model

Previously, Buehler et al. (2013) had used a large-scale AFLP genome scan (Herrmann et al. 2010; Poncet et al. 2010) to search for outlier loci under habitat-mediated selection in the alpine perennial plant *A. alpina* L., a close relative of the model organism *A. thaliana*. In two Alpine regions, the eastern Swiss and the French Alps, Buehler et al. (2013) had collected plants in three distinct habitat types: (1) rock/scree, (2) nutrient-rich and (3) moist. They applied rigorous selection criteria to detect outlier loci, especially parallel changes in habitat-related allele frequencies in both study regions and in the overall data set, and they excluded markers showing signals of spatial genetic structure. This analysis has led to the detection of one AFLP locus, EM74.7, as a consistent outlier locus among the 825 AFLP markers (Buehler et al. 2013). EM74.7 showed higher frequencies of the AFLP fragment in moist than in rock/scree or nutrient-rich habitats. Note that Poncet et al. (2010) performed an environmental association analysis, without referring to different habitat types, in which EM74.7 was not detected as a locus under selection.

### Sampling design of the independent validation data set

**Figure 2:**
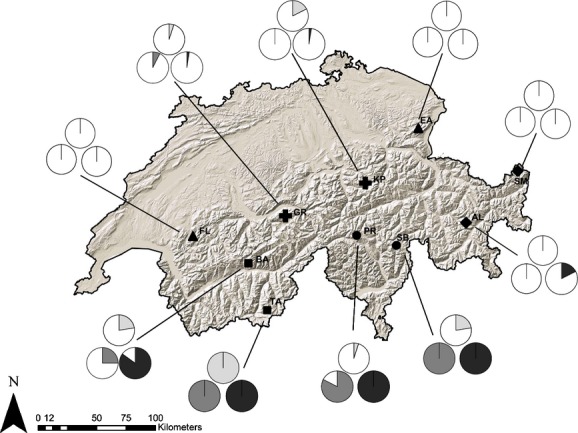
Sampling locations of *Arabis alpina* in five biogeographic regions of the Swiss Alps. Shown are pie charts of frequencies of SNaPshot®-inferred AFLP fragment presences at locus EM74.7 for three habitat types per location (moist: light gray; nutrient-rich: dark gray; rock/scree: black). Different symbols represent different biogeographic regions (▲ Prealps; **+** northern Alps; ♦ central eastern Alps; ▪ central western Alps; • southern Alps) and abbreviations denote sampling locations (FL, Flendruz; EA, Ebenalp; GR, Grindelwald; KP, Klausenpass; SM, Samnaun; AL, Albula; BA, Bachalp; TA, Täsch; PR, Piora; SB, San Bernardino).

For the independent data set of this study, we collected plants at ten sampling locations in summer 2010. Each sampling location consisted of three *A. alpina* populations (total of 30 populations) occurring in the three distinct habitat types of the original study (*i.e.,* rock/scree, nutrient-rich and moist; classified based on expert knowledge in the field; Fig. 2, Table S1 Supporting Information). The sampling locations were distributed in five biogeographic regions of the Swiss Alps (Hess et al. 1977): Prealps, northern Alps, central eastern Alps, central western Alps and southern Alps. Within each location, we searched for *A. alpina* occurrences in each of the three distinct habitat types, situated at 0.14–3.3 km distance. Buehler et al. (2012) showed that median pollen dispersal in *A. alpina* is about 20 m, but that exceptional long-distance dispersal of pollen up to 1 km is possible. The spatial separation of sampled habitat types within a location should therefore only allow for rare gene flow by pollen. No empirical data on seed dispersal distances are available, but the small seeds may likely be blown over several hundred meters, in particular across snow-covered landscape.

The rock/scree habitats were found in rock or scree fields along mountain slopes and were characterized by unstable substrate, low levels of organic matter, and irregular water availability. The nutrient-rich habitats were found in alpine pastures or underneath rocky cliffs, where nutrients naturally accumulate, and were characterized by high humus content and organic fertilization. The moist habitats were found along small alpine watercourses and were defined by high water availability and even occasional flooding (Buehler et al. 2013). At each location and in each population per habitat type, we sampled 20 individuals at distances of ≥ 2 m (600 individuals in total). Leaf material was dried in silica gel. DNA extraction was performed using the DNeasy 96 Plant Kit (Qiagen, Hilden, Germany) following the manufacturer's instructions.

### Genetic screening of the independent data set

Our focal AFLP outlier locus EM74.7 was sequenced following the method of Roden et al. (2009). In a BLAST search, the EM74.7 sequence appeared as a putative homolog to a SIT4 phosphatase-associated family protein of *A. thaliana* (GenBank accession no. NM_102783.4) and *A. lyrata* (GenBank accession no. XM_002890826.1), whose role in plant metabolism is unknown (Buehler et al. 2013). Primer pairs were designed in conserved upstream and downstream regions using PRIMER3 (Rozen and Skaletsky 2000), yielding the forward primer 5′ TCA CAC TAC CTT CTC TGG TTC C 3′, the reverse primer 5′ GCT TGG GTT GAG TGG AGA GA 3′ and a fragment length of 486 bp. To detect SNPs in the EM74.7 AFLP fragment underlying its presence/absence, we applied standard polymerase chain reaction (PCR) conditions for Sanger sequencing in 56 selected individuals of *A. alpina* from the original AFLP data set of Buehler et al. (2013).

**Figure 3:**
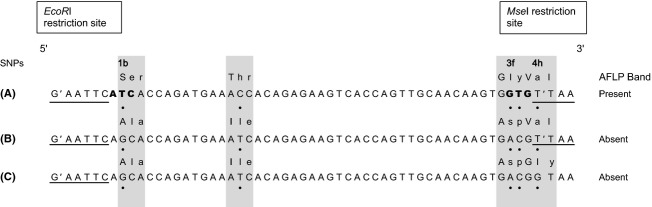
Alignment of sequences in *Arabis alpina* at locus EM74.7. Shown are sequences (A) in an *A. alpina* individual with the corresponding AFLP fragment present, and in two *A. alpina* individuals without the AFLP fragment, because of (B) polymorphisms in the selective bases and (C) a polymorphism in the *Mse*I restriction site. Restriction sites (*EcoR*I and *Mse*I) are underlined and marked at the cutting positions (′), the original selective bases are in bold, the nucleotide polymorphisms are marked (•), nucleotide polymorphisms leading to amino acid changes are highlighted with gray boxes, the corresponding amino acids are written above the nucleotide sequence, and the three SNPs used in analysis are labeled with marker names.

To screen the three selected SNPs causing AFLP fragment presence or absence (see Results), we used a fast and labor-effective SNaPshot® assay, which is a single-base primer extension method. To amplify the genomic region around the SNPs, we ran PCRs with the forward and reverse primers described above on all 600 samples of the independent data set. PCRs were carried out in a total volume of 10 *μ*L using 1x Multiplex PCR Kit (Qiagen, Hilden, Germany), 0.2 *μ*M of forward and reverse primers and approximately 1 ng of DNA template. Amplification took place on a Veriti Thermocycler (Applied Biosystems, Foster City, CA) with an initial polymerase activation at 95°C of 15 min, followed by 35 cycles of denaturation at 94°C for 30 s, primer annealing at 56°C for 90 s and 72°C for 60 s and followed by a final extension at 72°C for 10 min. PCR products were purified by incubating 1 *μ*L of ExoSAP–IT® (USB, Cleveland, OH) in 5 *μ*L PCR product at 37°C for 15 min followed by 80°C for 15 min for enzyme inactivation. Primers for SNaPshot® reactions were designed to anneal in the flanking regions directly adjacent to either of the three SNPs detected (1b, 3f and 4 h in Fig. 3). BATCHPRIMER3 (You et al. 2008) was used to design primers on sense or anti-sense DNA strands with an annealing temperature of 50–60°C. To test for possible hairpin structures and primer dimers, AUTODIMER (Vallone and Butler 2004) was used. Poly-(T) tails were added to primers to increase the length of the extension products allowing for multiplexing. The selected HPLC-purified primers were combined in a multiplex reaction in which PCR products ranged from 24 to 42 bp (Table 1). Single-base extension (SBE) was performed using 2.5 *μ*L SNaPshot® ready reaction mix (Applied Biosystems), 0.2 *μ*M of each primer and 1.5 *μ*L of the above purified PCR product in 6 *μ*L total volume. SBE reactions were carried out on a Veriti Thermocycler (Applied Biosystems) with 27 cycles comprising of 96°C for 10 s, 52°C for 5 s and 60°C for 30 s. After SBE reaction, we performed a post-extension treatment to remove unincorporated nucleotides causing high background fluorescence signals, using 5 *μ*L PCR product treated with 0.5 *μ*L of shrimp alkaline phosphatase (SAP; USB Corporation, Cleveland, OH) at 60°C for 30 min followed by incubation at 80°C for 15 min for enzyme inactivation. The SBE products were run on an ABI 3130 capillary sequencer (Applied Biosystems) by mixing 0.5 *μ*L SBE product with 9 *μ*L Hi-Di Formamide and 0.5 *μ*L GeneScan 120LIZ internal size standard (Applied Biosystems). Results were analyzed with GENEMAPPER 3.7 (Applied Biosystems). We verified the confidence of the multiplex SNaPshot® assay on 20 previously sequenced and AFLP-genotyped individuals from the original AFLP data set.

**Table 1:** Single-nucleotide polymorphisms (SNPs) at locus EM74.7 in *Arabis alpina* in the independent data set of the Swiss Alps. Given are the SNP marker names, nucleotide base changes, haplotype frequencies, single-base extensions (SBE) primers including the poly(T) tail and primer length (bp)

Marker	SNP	Haplotype frequency	SBE extension primer	Size [bp]
1b	G	0.733	GATAAAAAGAGTGCAGAGAATTCA	24
	T	0.267		
3f	A	0.733	(T)_26_CCAGTTGCAACAAGTG	42
	G	0.267		
4 h	A	1.000	(T)_17_GAGGTCTCAGTGGTTTTA	35
	C	0.000		

### Validation of allele distribution model

Haplotype frequencies per population in the independent data set were estimated using GENEPOP 4.0 (Rousset 2008). General linear models were used to test the effects of habitat type and biogeographic region on arcsine-transformed haplotype frequencies using SPSS 17.0 (SPSS, Chicago, IL). Habitat type was treated as a fixed factor and biogeographic region and location were treated as random factors, with location nested within region. The interaction of habitat and region was also tested. We expected EM74.7 to show the same genotype association with habitat types in the independent data set as that found in the original study (Buehler et al. 2013).

### Additional molecular analyses

As we were not able to validate the original allele distribution model in our independent data set (see Results), we explored several causes for this failure (see Introduction). One of these causes refers to technical problems with AFLP genotyping. To investigate this potential cause, we went back to the original samples (Herrmann et al. 2010; Poncet et al. 2010) to check for discrepancies between AFLP scoring and SNP calling. We used the same SNaPshot® assay as described above to genotype all samples from the French and Swiss Alps used in the original data set by Buehler et al. (2013; *N* = 699), transformed the resulting haplotypes into AFLP fragment presences or absences and compared these *in silico* AFLP patterns with the original AFLP data. We could thus assess whether an AFLP fragment at EM74.7 was truly present, falsely present, or truly absent. We then visually inspected the AFLP fragment patterns of EM74.7 using the overlay function of Genemapper 3.7 (Applied Biosystems). Finally, the scorings of EM74.7 in the original AFLP data set used by Buehler et al. (2013) to detect outlier loci were modified according to these SNaPshot® results (see Results), and a new outlier analysis was carried out with Dfdist (Beaumont and Nichols 1996; Beaumont and Balding 2004) on these original samples of the French Alps, of the Swiss Alps and of the combined data sets using the same parameters as in Buehler et al. (2013) to check whether EM74.7 remained an outlier locus.

## Results

### Validation of outlier locus and allele distribution model

The sequences of 56 *A. alpina* individuals from the original AFLP data set of Buehler et al. (2013) showed several polymorphisms within the sequence of EM74.7 (Fig. 3; sequence variants are available from GenBank accession nos. HM594277–HM594279). At the 5′ end, there was a polymorphism in one of the AFLP selective bases (T/G). At the 3′ end, there was a two-base pair polymorphism in the selective bases (A/G, C/T). These polymorphisms were linked, meaning that all individuals with fragment presence displayed the same mutations (1b and 3f in Fig. 3). A few individuals also had a polymorphism in the *Mse*I restriction site (T/G; 4 h in Fig. 3). All of these polymorphisms were accounting for AFLP fragment presence or absence. There was an additional polymorphism within the fragment sequence (T/C; Fig. 3).

In the independent validation data set, we amplified the three sequence-characterized mutations underlying the AFLP fragment presence or absence using SNaPshot®. The electrophoretic mobility of the SBE products as determined by the automated sequencer was slightly different from the actual size of the products, but the spacing between SBE primers was large enough to obtain clearly separated peaks. Two loci, 1b and 3f, were biallelic and showed similar haplotype frequencies in all samples due to linkage (T: H_E_ = 0.267 and G: H_E_ = 0.267; Table 1). Locus 4 h, however, was monomorphic in the independent data set (T: H_E_ = 1.000; Table 1) and not linked with SNP loci 1b and 3f.

**Figure 4:**
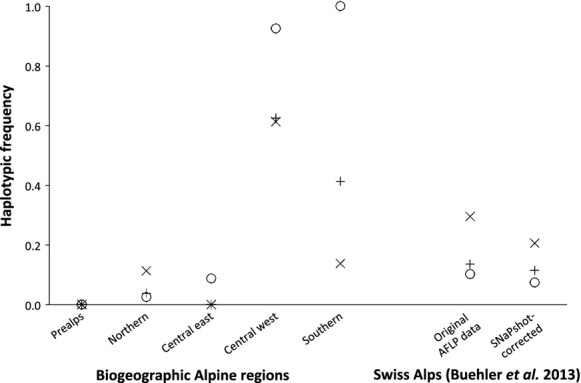
Haplotype frequencies of SNaPshot®-inferred AFLP fragment presences at locus EM74.7 in different habitat types in five biogeographic Alpine regions of Switzerland (Prealps, northern Alps, central eastern Alps, central western Alps, southern Alps). For reference, habitat-specific fragment presences are also given for the original and the SNaPshot®-corrected AFLP data set of Swiss samples published by Buehler et al. (2013). Habitat types: rock/scree (o, dashed line); nutrient-rich (+, dotted line); moist (x, solid line).

The general linear model analysis did not detect a significant effect of habitat type on haplotype frequencies at EM74.7 (*P* = 0.191; Table 2). Instead, the effect of bio-graphic regions was significant (*P* = 0.038; Table 2). Strikingly, the regions central western Alps and southern Alps showed strong deviations in haplotype frequencies from all other regions (Fig. 4). The interaction of habitat and region on haplotype frequencies at EM74.7, however, was not significant (*P* = 0.166; Table 2).

**Table 2:** General linear model of haplotype frequency at locus EM74.7 in different habitat types (rock/scree; nutrient-rich; moist) of *Arabis alpina* with habitat type (fixed factor), biogeographic region (random factor), location nested within region (random factor) and habitat × biogeographic region interaction

Source	df	MS	*F*	*P*
Habitat	2	0.329	2.051	0.191
Biogeographic region	4	1.556	4.865	0.038*
Location [Biogeographic region]	5	0.243	2.905	0.071
Habitat × biogeographic region	8	0.160	1.912	0.166

df, degrees of freedom; MS, mean sum of squares.

* *P* < 0.05.

### Additional molecular analyses

The multiplex SNaPshot® assay of the original sample set gave scorable profiles for 561 samples: several samples had to be excluded, because SNaPshot® reactions did not amplify or could not be reliably scored. The SNaPshot® analysis of EM74.7 showed that of these 561 samples, 17 (3%) were incorrectly genotyped in the original AFLP analysis of Poncet et al. (2010) and Herrmann et al. (2010). This means that of 92 individuals that were scored to have an AFLP fragment at EM74.7, 17 should have been scored as having no fragment based on the SNaPshot® information. This error rate was only slightly higher than the overall original AFLP error rate of 1.2% given in Herrmann et al. (2010). In the Swiss range of the original data set, 13 individuals were falsely scored (six from moist sites, five from rock/scree, two from nutrient-rich habitats), whereas four plants were incorrectly assigned in the original French data set (two each from rock/scree and nutrient-rich habitats, respectively).

**Figure 5:**
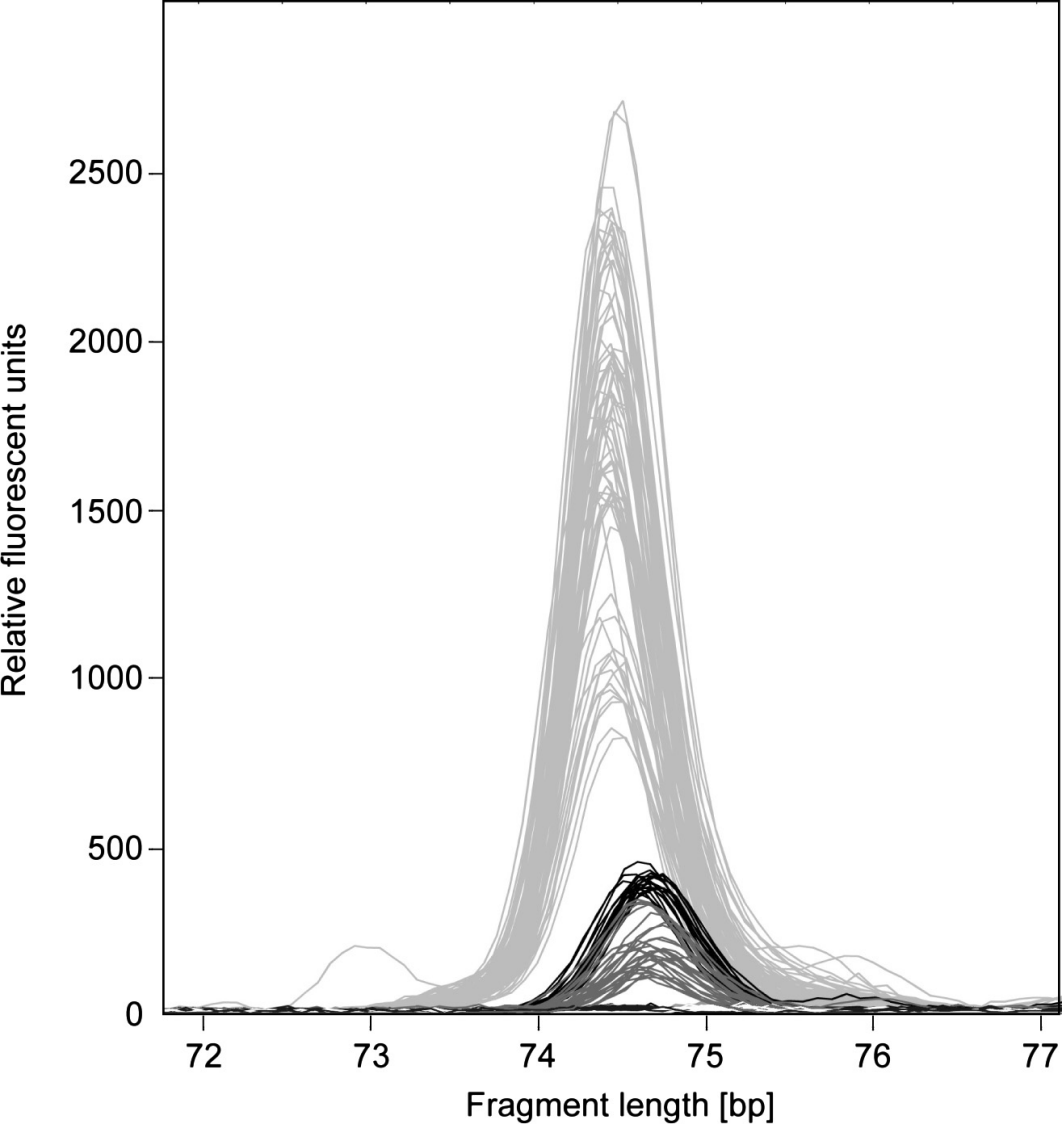
AFLP electropherograms associated with EM74.7 in *Arabis alpina* from the French and Swiss Alps, originally published by Buehler et al. (2013). Light gray peaks represent the true AFLP fragment, the remaining peaks with a slightly higher amplicon size represent another, uncharacterized fragment of similar size. The higher peaks of the latter fragment were incorrectly scored as being present at EM74.7 (black), the lower peaks were scored as being absent (dark gray).

The visual comparison of the original AFLP peaks showed that these observed discrepancies likely resulted from size homoplasy, that is*,* two different fragments were combined into one bin for EM74.7 during manual AFLP scoring. The two fragments could visually be distinguished upon close inspection, one with a mean fragment size of 74.45 bp ± 0.04 (SD) and the other with a mean of 74.68 bp ± 0.07 (Fig. 5), the former representing the true fragment used in the AFLP analysis. As the peaks of the longer fragments showed considerably lower peak heights than those at EM74.7*,* they were mostly filtered out of the data set because of the rigorous selection criteria applied by Herrmann et al. (2010). In some cases, however, these false fragments had peak heights that were slightly higher than the peak height threshold applied in Herrmann et al. (2010). They were thus kept in the data set and scored as fragment presence at EM74.7, even though the true fragment was absent.

After correcting the original AFLP data set of Buehler et al. (2013) for SNaPshot®-based fragment presence/absence at EM74.7, the dfdist outlier analysis still detected this locus as an outlier in the original sample from the French Alps and in the combined data set (French and Swiss samples). However, it was no longer considered an outlier in the original sample from the Swiss Alps, the region where we performed the present validation test.

## Discussion

### Failed validation of outlier locus and allele distribution model

The use of environmental association analysis in combination with outlier analysis to detect loci potentially of adaptive relevance to environmental factors is widespread (Schoville et al. 2012). Still, the extent to which identified outlier loci and corresponding allele distribution models can be verified remains largely unexplored (Wiener et al. 2011). In the present study, we attempted to validate a known allele distribution at an outlier locus that had previously been identified in *A. alpina* by testing it in an independent and replicated data set. In the former study of Buehler et al. (2013), AFLP fragments at locus EM74.7 had shown a higher association with moist habitat types than with nutrient-rich or rock/scree habitats in the eastern Swiss and French Alps. The results of the present study, however, did not support this pattern in a validation data set from across the Swiss Alps: no significant correlation between frequencies of AFLP fragment presence (based on SNaPshot® haplotypes) and habitat types could be found. We therefore failed to validate the allele distribution model at EM74.7 found in the original study in the validation dataset of the Swiss Alps. However, one should note that a validation in the French Alps as included in the original study by Buehler et al. (2013) would be a valuable addition to the study presented here. Similar to our results, Nachmann et al. (2003) investigated a candidate gene for coat color in pocket mice and failed to find concordance of the environment x allele relationship among populations from Arizona and New Mexico.

Below, we evaluate and discuss several explanations for our failure to validate the differential distribution of alleles among habitat types in the independent data set of the present study.

### Potential causes for the failure of validation

First, local adaptation could cause EM74.7 to be indicative of habitat-mediated selection only in the regions sampled for the original AFLP data set by Poncet et al. (2010) and Herrmann et al. (2010). Plant populations often adapt to local environmental conditions driving the evolution of local genotypes or ecotypes (Joshi et al. 2001; Hoffmann and Willi 2008). Moreover, selection can only act on alleles, that is*,* genetic variation, present in a given population. The fact that we found a completely different pattern of haplotype frequencies at locus EM74.7 in the sampling locations in the southern Swiss Alps could be considered an indication that this locus represents a truly local case of adaptation. The regionally divergent pattern found in the present study may have been accentuated by the mating system of *A. alpina* owing to its high selfing rate (Ansell et al. 2008; Tedder et al. 2011; Buehler et al. 2012), so that a particular allele may rapidly spread once established and hence mask adaptive patterns. In order to confirm such a hypothesis of local adaptation, more locations on a small spatial scale would have to be sampled in the original study areas in the Swiss as well as the French Alps (Buehler et al. 2013), which the present study did not attempt to validate. Furthermore, classical transplant experiments could be performed to effectively prove that local adaptation is causing habitat-mediated selection (Holderegger et al. 2008).

Second, it is well known that geographic population structure affects outlier detection, causing false positives (Excoffier et al. 2009; Nosil et al. 2009). In fact, we detected a significant association of allele frequencies at EM74.7 with biogeographic regions in the present study (Table 2). This finding suggests that geography was a strong driver of population divergence at EM74.7 at least within the Swiss range of *A. alpina*. An association of allele frequencies with geographic area can arise from restricted gene flow, bottlenecks, range contraction, or expansions during glaciation or by a combination of these factors (Templeton et al. 1995). Alpine ecosystems are known to show distinct population genetic structure as populations often diverged in separate glacial refugia and came into secondary contact through re-expansion (Schönswetter et al. 2005; Alvarez et al. 2009; Thiel-Egenter et al. 2011). Therefore, Buehler et al. (2013) had applied rigorous selection criteria also controlling for geographic structure in the original outlier analysis: EM74.7 had been a consistent outlier across two Alpine regions and had shown no genetic structure. However, the phylogeographic history of species is complex and in most cases unknown (Excoffier et al. 2009). It is likely that the way in which genetic structure had been tested in *A. alpina* by Buehler et al. (2013) did not adequately reflect the actual genetic structure across the entire Swiss Alps, which is the area considered in the present validation study. Thus, it is plausible that EM74.7 was not under selection because of the confounding effects of spatial genetic structure. Similarly to our results, Shikano et al. (2010) explored the effect of habitat type (marine vs. freshwater) and geographic area on population divergence in nine-spined sticklebacks and found that most loci detected to be under selection for salinity were in fact associated with geography.

Third, EM74.7 could still be under adaptive divergence, but the regional differences in allele frequencies were attributed to ecological gradients that were not tested for in the original and the validation data set. This would be the case if such an unmeasured, but relevant environmental factor was correlated with habitat types in the original sample tested by Buehler et al. (2013), but uncorrelated with habitat types in the present study. Especially temperature and precipitation have been shown to be determinants of allele distribution in *A. alpina* and other alpine plants (Manel et al. 2010, 2012; Poncet et al. 2010; Bothwell et al. 2013). These and other climatic factors are likely to be intermingled with geographic area. Therefore, they could explain the discrepancies in allele frequencies found between the original and the present studies if the latter were triggered by unaccounted environmental gradients.

Fourth, a technical issue has contributed to EM74.7 being a false outlier. Our additional laboratory analysis with SNP genotyping revealed that there was a certain discrepancy between the original AFLP and the SNaPshot®-derived data sets. In the samples from the eastern Swiss Alps, two fragments of slightly different sizes had been combined into the same bin in the original AFLP data set of Buehler et al. (2013; Fig. 5). This was, however, only rarely the case for the samples from the French Alps. In fact, when the original data set was adjusted according to the results of the SNaPshot®-analysis, which changed the average allele frequency of EM74.7 primarily in moist populations from the original Swiss range (from 0.310 to 0.225; Fig. 4), this locus was no longer detected as a habitat-mediated outlier in the Swiss Alps. In turn, EM74.7 retained its outlier status in the original data set of the French Alps and in the combined dataset of the Swiss and French Alps. These results confirm that homoplasy is indeed an issue in the analysis of AFLP data (Arrigo et al. 2009). Different fragments may be very similar in size and thus be scored as belonging to the same marker (Vekemans et al. 2002; Meudt and Clarke 2007). Defining bin width is thus a crucial step in AFLP scoring. In general, we suggest selecting narrow bins, as the precision of automated capillary sequencers in estimating fragment sizes is generally high, leading to minimal variance among truly homologous fragments (Wenz et al. 1998), and hence reducing the degree of homoplasy in an AFLP data set.

## Conclusions

In the present study, we demonstrate that a validation approach with an independent data set, that is*,* replication in one or several alternative region(s), helps to consolidate outlier loci and corresponding allele distribution models or, in the case validation fails, to unravel possible causes for the inconsistencies between original and validation data sets. In the present study, we developed several potential explanations for validation failure and conclude that, in the case of the habitat-mediated outlier locus EM74.7 of *A. alpina,* geographic population structure as well as technical issues were the most plausible. Further studies using additional, codominant markers, for example, nuclear microsatellites (Buehler et al. 2011), could elucidate whether inbreeding or demographic processes underlie the regional pattern observed.

As outlier locus detection and environmental association studies are often carried out in single, nonreplicated areas (Buerkle et al. 2011) and because proof of the functional adaptive relevance of outlier loci to environmental factors is seldom given in molecular biology or transplant/common garden experiments (Manel et al. 2010), at least in nonmodel organisms, we advocate that using independent data sets to test outlier loci and their environmental association is an adequate tool in ecological genomics. Validation provides a better understanding of inferred signatures of selection and adds confidence to outlier loci detected, and it may help avoid outlier loci which turn out to be false positives.

## Data Accessibility

DNA sequences: GenBank accessions: HM594277–HM594279.

## Supporting Information

**Table S1**. Sample locations and AFLP fragment frequencies.
