# Supplementary material for: Validation of outlier loci through replication in independent data sets: a test on Arabis alpina
Source: Ecol Evol. 2014 Oct 24;4(22):4296–306. doi: 10.1002/ece3.1300 (PMC4267868; doi:10.1002/ece3.1300)
Supplement: Supplementary file 1 — Table S1. Sample locations and AFLP fragment frequencies. [file ece30004-4296-SD1.docx]

**Supplementary Table S1** AFLP fragment presence/absence patterns of 600 *Arabis alpina* individuals of the independent validation data set occurring in different habitat types in the Swiss Alps. Shown are the biogeographic region of each population, habitat type, latitude and longitude and the allele ratio for presence and absence of the AFLP fragment per population.

| Region | Population | Habitat | Latitude N | Longitude E |  | Absence | Presence |
| --- | --- | --- | --- | --- | --- | --- | --- |
| Central eastern | Samnaun | Moist | 46.92474 | 10.36237 |  | 0.000 | 1.000 |
|  |  | Nutrient-rich | 46.92964 | 10.36370 |  | 0.000 | 1.000 |
|  |  | Rock/Scree | 46.91433 | 10.35735 |  | 0.000 | 1.000 |
|  | Albula | Moist | 46.58626 | 9.85532 |  | 0.000 | 1.000 |
|  |  | Nutrient-rich | 46.58308 | 9.84279 |  | 0.000 | 1.000 |
|  |  | Rock/Scree | 46.57976 | 9.82256 |  | 0.175 | 0.825 |
| Central western | Bachalp | Moist | 46.35745 | 7.68919 |  | 0.225 | 0.775 |
|  |  | Nutrient-rich | 46.36893 | 7.68899 |  | 0.250 | 0.750 |
|  |  | Rock/Scree | 46.34563 | 7.68469 |  | 0.850 | 0.150 |
|  | Täsch | Moist | 46.05950 | 7.81365 |  | 1.000 | 0.000 |
|  |  | Nutrient-rich | 46.05830 | 7.81349 |  | 1.000 | 0.000 |
|  |  | Rock/Scree | 46.05159 | 7.82825 |  | 1.000 | 0.000 |
| Northern | Grindelwald | Moist | 46.67537 | 8.06686 |  | 0.050 | 0.950 |
|  |  | Nutrient-rich | 46.66198 | 8.05357 |  | 0.075 | 0.925 |
|  |  | Rock/Scree | 46.67898 | 8.06902 |  | 0.025 | 0.975 |
|  | Klausenpass | Moist | 46.87473 | 8.83370 |  | 0.175 | 0.825 |
|  |  | Nutrient-rich | 46.87299 | 8.82855 |  | 0.000 | 1.000 |
|  |  | Rock/Scree | 46.87538 | 8.84083 |  | 0.025 | 0.975 |
| Prealps | Ebenalp | Moist | 47.26984 | 9.40163 |  | 0.000 | 1.000 |
|  |  | Nutrient-rich | 47.27743 | 9.39822 |  | 0.000 | 1.000 |
|  |  | Rock/Scree | 47.27972 | 9.39766 |  | 0.000 | 1.000 |
|  | Flendruz | Moist | 46.51126 | 7.16200 |  | 0.000 | 1.000 |
|  |  | Nutrient-rich | 46.51891 | 7.15386 |  | 0.000 | 1.000 |
|  |  | Rock/Scree | 46.51129 | 7.14422 |  | 0.000 | 1.000 |
| Southern | Piora | Moist | 46.54517 | 8.72637 |  | 0.050 | 0.950 |
|  |  | Nutrient-rich | 46.53968 | 8.74504 |  | 0.825 | 0.175 |
|  |  | Rock/Scree | 46.53089 | 8.74916 |  | 1.000 | 0.000 |
|  | San Bernardino | Moist | 46.47517 | 9.14580 |  | 0.225 | 0.775 |
|  |  | Nutrient-rich | 46.49896 | 9.17070 |  | 0.000 | 1.000 |
|  |  | Rock/Scree | 46.48693 | 9.15717 |  | 1.000 | 0.000 |
